# 4-Meth­oxy-3-(trifluoro­meth­yl)aniline

**DOI:** 10.1107/S160053681200030X

**Published:** 2012-01-14

**Authors:** Jian-Ling He

**Affiliations:** a College of Chemical and Biological Engineering, Yancheng Institute of Technology, Yinbing Road No. 9 Yancheng, Yancheng 224051, People’s Republic of China

## Abstract

In title compound, C_8_H_8_F_3_NO, the meth­oxy group is inclined at 8.7 (4)° to the benzene ring plane. The crystal structure is stabilized by inter­molecular N—H⋯F, N—H⋯N and C—H⋯F hydrogen-bonding inter­actions.

## Related literature

The title compound is an inter­mediate in the synthesis of trifluoro­methyl-containing phthalic acid diamides, which are effective pesticides. For the preparation, see: Feng & Li (2010 ▶). For the crystal structure of a closely related compound, see: Crampton *et al.* (2006 ▶).
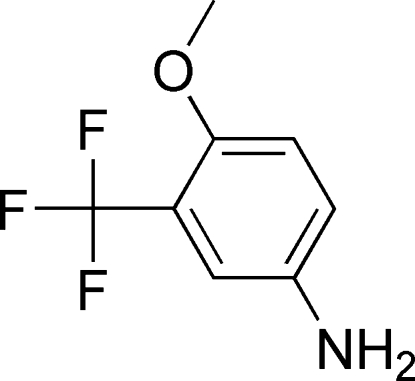


## Experimental

### 

#### Crystal data

C_8_H_8_F_3_NO
*M* *_r_* = 191.15
Orthorhombic, 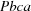

*a* = 5.4140 (11) Å
*b* = 14.880 (3) Å
*c* = 21.304 (4) Å
*V* = 1716.3 (6) Å^3^
*Z* = 8
Mo *K*α radiation
μ = 0.14 mm^−1^
*T* = 293 K
0.10 × 0.10 × 0.10 mm

#### Data collection

Enraf–Nonius CAD-4 diffractometer
Absorption correction: ψ scan (North *et al.*, 1968 ▶) *T*_min_ = 0.986, *T*_max_ = 0.986
1722 measured reflections
1722 independent reflections
1389 reflections with *I* > 2σ(*I*)

#### Refinement

*R*[*F* ^2^ > 2σ(*F* ^2^)] = 0.059
*wR*(*F* ^2^) = 0.162
*S* = 1.13
1722 reflections
118 parameters
H-atom parameters constrained
Δρ_max_ = 0.20 e Å^−3^
Δρ_min_ = −0.19 e Å^−3^

### 

Data collection: *CAD-4 Software* (Enraf–Nonius, 1985 ▶); cell refinement: *CAD-4 Software*; data reduction: *XCAD4* (Harms & Wocadlo, 1995 ▶); program(s) used to solve structure: *SHELXS97* (Sheldrick, 2008 ▶); program(s) used to refine structure: *SHELXL97* (Sheldrick, 2008 ▶); molecular graphics: *SHELXTL* (Sheldrick, 2008 ▶); software used to prepare material for publication: *SHELXTL*.

## Supplementary Material

Crystal structure: contains datablock(s) I, global. DOI: 10.1107/S160053681200030X/pv2499sup1.cif

Structure factors: contains datablock(s) I. DOI: 10.1107/S160053681200030X/pv2499Isup2.hkl

Supplementary material file. DOI: 10.1107/S160053681200030X/pv2499Isup3.cml

Additional supplementary materials:  crystallographic information; 3D view; checkCIF report

## Figures and Tables

**Table 1:** Hydrogen-bond geometry (Å, °)

*D*—H⋯*A*	*D*—H	H⋯*A*	*D*⋯*A*	*D*—H⋯*A*
C1—H1*C*⋯F2^i^	0.96	2.52	3.292 (3)	138
N—H0*A*⋯F1^ii^	0.86	2.44	3.242 (2)	155
N—H0*B*⋯N^iii^	0.86	2.47	3.245 (3)	150

Symmetry codes: (i) 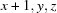; (ii) 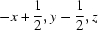; (iii) 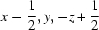.

**Fig. 1.**
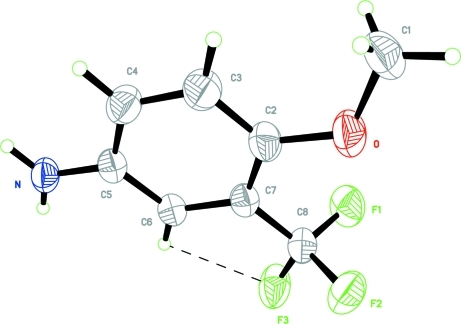
The molecular structure of the title molecule with the atom-numbering scheme. Displacement ellipsoids are drawn at the 50% probability level.

**Fig. 2.**
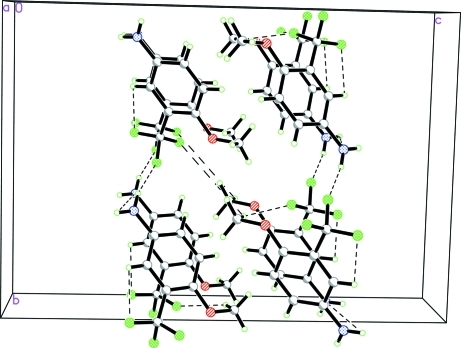
A packing diagram of the title compound showing hydrogen bonds dashed lines.

## supplementary crystallographic information

### Comment

The tittle compound (Fig. 1) is used as an important intermediate in the synthesis of trifluoromethyl-containing phthalic acid compounds which are recognized as effective pesticides (Feng & Li, 2010). In the title molecule, the N, O and C8 atoms bonded to the central benzene ring (C2–C7) lie in its plane with methoxy group (O/C1) oriented at 8.7 (4) ° with respect to the benzene ring plane. There is an intramolecular interaction C6—H6A···F3 which stabilizes the molecular structure of the title compound. The crystal structure is stabilized by N—H0A···F1, N—H0B···N and C1—H1C···F2 intermolecular hydrogen bonding interactions. The bond distances and bond angles in the title compound agree with the corresponding bond distances and bond angles reported in a closely related compound (Crampton *et al.*, 2006).

### Experimental

The title compound was prepared by a method reported in the literature (Feng & Li, 2010)). A solution of 4-amino-2-(trifluoromethyl)phenol (2 g, 11.3 mmol) in dichloromethane (20 ml) was added slowly to a solution of sodium hydride (0.33 g, 13.6 nmol) in an ice bath. After stirring for 6 h iodomethane (4.8 g, 33.9 mmol) was added slowly in 1 h. After stirring for 48 h at room tempeature, the solvent was evaporated on a rotary evaporator yielding the title compound. Colorless blocks of the title compound were grown in ethanol (20 ml) by slow evaporation of the solvent at room temperature in about 7 days.

### Refinement

The H atoms were positioned geometrically and constrained to ride on their parent atoms, with N—H = 0.86 Å and C—H = 0.93 and 0.96 Å for aryl and alky H-atoms, respectively, with *U*_iso_(H) = *xU*_eq_(C/N), where *x* = 1.2 for aromatic and amino H, and *x* = 1.5 for aryl H atoms.

### Crystal data

**Table:**

C_8_H_8_F_3_NO	*F*(000) = 784
*M**_r_* = 191.15	*D*_x_ = 1.480 Mg m^−^^3^
Orthorhombic, *P**b**c**a*	Mo *K*α radiation, λ = 0.71073 Å
Hall symbol: -P 2ac 2ab	Cell parameters from 25 reflections
*a* = 5.4140 (11) Å	θ = 3.3–20.0°
*b* = 14.880 (3) Å	µ = 0.14 mm^−^^1^
*c* = 21.304 (4) Å	*T* = 293 K
*V* = 1716.3 (6) Å^3^	Prism, colorless
*Z* = 8	0.10 × 0.10 × 0.10 mm

### Data collection

**Table:**

Enraf–Nonius CAD-4 diffractometer	1722 independent reflections
Radiation source: fine-focus sealed tube	1389 reflections with *I* > 2σ(*I*)
graphite	*R*_int_ = 0.0000
Detector resolution: 28.5714 pixels mm^-1^	θ_max_ = 26.4°, θ_min_ = 3.3°
ω/2θ scans	*h* = 0→6
Absorption correction: ψ scan (North *et al.*, 1968)	*k* = 0→18
*T*_min_ = 0.986, *T*_max_ = 0.986	*l* = 0→26
1722 measured reflections	

### Refinement

**Table:**

Refinement on *F*^2^	Primary atom site location: structure-invariant direct methods
Least-squares matrix: full	Secondary atom site location: difference Fourier map
*R*[*F*^2^ > 2σ(*F*^2^)] = 0.059	Hydrogen site location: inferred from neighbouring sites
*wR*(*F*^2^) = 0.162	H-atom parameters constrained
*S* = 1.13	*w* = 1/[σ^2^(*F*_o_^2^) + (0.074*P*)^2^ + 0.3348*P*] where *P* = (*F*_o_^2^ + 2*F*_c_^2^)/3
1722 reflections	(Δ/σ)_max_ < 0.001
118 parameters	Δρ_max_ = 0.20 e Å^−^^3^
0 restraints	Δρ_min_ = −0.19 e Å^−^^3^

### Special details

**Table:**

Geometry. All e.s.d.'s (except the e.s.d. in the dihedral angle between two l.s. planes) are estimated using the full covariance matrix. The cell e.s.d.'s are taken into account individually in the estimation of e.s.d.'s in distances, angles and torsion angles; correlations between e.s.d.'s in cell parameters are only used when they are defined by crystal symmetry. An approximate (isotropic) treatment of cell e.s.d.'s is used for estimating e.s.d.'s involving l.s. planes.
Refinement. Refinement of *F*^2^ against ALL reflections. The weighted *R*-factor *wR* and goodness of fit *S* are based on *F*^2^, conventional *R*-factors *R* are based on *F*, with *F* set to zero for negative *F*^2^. The threshold expression of *F*^2^ > σ(*F*^2^) is used only for calculating *R*-factors(gt) *etc*. and is not relevant to the choice of reflections for refinement. *R*-factors based on *F*^2^ are statistically about twice as large as those based on *F*, and *R*- factors based on ALL data will be even larger.

### Fractional atomic coordinates and isotropic or equivalent isotropic displacement parameters (Å^2^)

**Table:**

	*x*	*y*	*z*	*U*_iso_*/*U*_eq_	
N	0.0910 (3)	0.09833 (11)	0.20799 (9)	0.0642 (5)	
H0A	0.1826	0.0524	0.2003	0.077*	
H0B	0.0020	0.0998	0.2414	0.077*	
F3	−0.3126 (4)	0.39455 (11)	0.21116 (7)	0.1021 (6)	
F2	−0.3656 (3)	0.42452 (10)	0.11387 (8)	0.0906 (5)	
C7	−0.0486 (4)	0.32133 (13)	0.13969 (9)	0.0548 (5)	
F1	−0.0537 (3)	0.47609 (9)	0.16277 (8)	0.0924 (6)	
O	0.0805 (4)	0.39518 (12)	0.04733 (8)	0.0872 (6)	
C6	−0.0515 (4)	0.24725 (13)	0.17919 (10)	0.0550 (5)	
H6A	−0.1479	0.2487	0.2153	0.066*	
C5	0.0868 (4)	0.17085 (13)	0.16592 (9)	0.0557 (5)	
C8	−0.1937 (4)	0.40274 (15)	0.15659 (10)	0.0643 (6)	
C2	0.0907 (4)	0.32022 (15)	0.08453 (10)	0.0653 (6)	
C4	0.2268 (5)	0.17158 (16)	0.11146 (11)	0.0719 (7)	
H4A	0.3216	0.1214	0.1016	0.086*	
C3	0.2297 (5)	0.24452 (18)	0.07140 (11)	0.0767 (7)	
H3A	0.3260	0.2427	0.0353	0.092*	
C1	0.2463 (6)	0.4017 (2)	−0.00398 (11)	0.0865 (8)	
H1A	0.2177	0.4570	−0.0260	0.130*	
H1B	0.2203	0.3520	−0.0319	0.130*	
H1C	0.4131	0.4005	0.0113	0.130*	

### Atomic displacement parameters (Å^2^)

**Table:**

	*U*^11^	*U*^22^	*U*^33^	*U*^12^	*U*^13^	*U*^23^
N	0.0752 (12)	0.0474 (10)	0.0699 (11)	0.0025 (8)	0.0031 (9)	0.0081 (8)
F3	0.1383 (15)	0.0767 (10)	0.0912 (11)	0.0339 (10)	0.0413 (10)	0.0151 (8)
F2	0.0887 (10)	0.0811 (11)	0.1018 (11)	0.0177 (8)	−0.0227 (9)	0.0065 (8)
C7	0.0605 (11)	0.0477 (11)	0.0562 (11)	−0.0037 (9)	−0.0035 (10)	0.0018 (8)
F1	0.1106 (12)	0.0541 (8)	0.1124 (12)	−0.0104 (8)	−0.0073 (9)	−0.0123 (7)
O	0.1094 (14)	0.0758 (11)	0.0763 (11)	0.0142 (10)	0.0150 (10)	0.0304 (9)
C6	0.0620 (12)	0.0492 (11)	0.0539 (11)	−0.0033 (9)	0.0009 (10)	0.0014 (8)
C5	0.0625 (12)	0.0482 (11)	0.0563 (11)	−0.0046 (9)	−0.0058 (10)	0.0021 (8)
C8	0.0757 (15)	0.0539 (12)	0.0632 (12)	0.0042 (10)	0.0003 (11)	0.0069 (10)
C2	0.0782 (14)	0.0604 (13)	0.0572 (12)	0.0005 (11)	0.0006 (11)	0.0119 (10)
C4	0.0852 (16)	0.0629 (14)	0.0674 (13)	0.0141 (12)	0.0126 (13)	0.0032 (10)
C3	0.0922 (17)	0.0765 (16)	0.0614 (13)	0.0136 (13)	0.0179 (13)	0.0116 (11)
C1	0.0955 (18)	0.0942 (19)	0.0697 (15)	−0.0139 (15)	0.0077 (15)	0.0258 (14)

### Geometric parameters (Å, °)

**Table:**

N—C5	1.403 (3)	C6—C5	1.390 (3)
N—H0A	0.8600	C6—H6A	0.9300
N—H0B	0.8600	C5—C4	1.386 (3)
F3—C8	1.335 (3)	C2—C3	1.383 (3)
F2—C8	1.342 (3)	C4—C3	1.381 (3)
C7—C6	1.387 (3)	C4—H4A	0.9300
C7—C2	1.396 (3)	C3—H3A	0.9300
C7—C8	1.488 (3)	C1—H1A	0.9600
F1—C8	1.335 (3)	C1—H1B	0.9600
O—C2	1.369 (3)	C1—H1C	0.9600
O—C1	1.418 (3)		
			
C5—N—H0A	120.0	F1—C8—C7	112.9 (2)
C5—N—H0B	120.0	F2—C8—C7	113.50 (19)
H0A—N—H0B	120.0	O—C2—C3	124.6 (2)
C6—C7—C2	120.48 (19)	O—C2—C7	117.1 (2)
C6—C7—C8	119.62 (19)	C3—C2—C7	118.27 (19)
C2—C7—C8	119.89 (18)	C3—C4—C5	122.0 (2)
C2—O—C1	118.4 (2)	C3—C4—H4A	119.0
C7—C6—C5	121.37 (19)	C5—C4—H4A	119.0
C7—C6—H6A	119.3	C4—C3—C2	120.6 (2)
C5—C6—H6A	119.3	C4—C3—H3A	119.7
C4—C5—C6	117.27 (18)	C2—C3—H3A	119.7
C4—C5—N	122.13 (19)	O—C1—H1A	109.5
C6—C5—N	120.53 (19)	O—C1—H1B	109.5
F3—C8—F1	105.23 (19)	H1A—C1—H1B	109.5
F3—C8—F2	106.14 (19)	O—C1—H1C	109.5
F1—C8—F2	105.25 (18)	H1A—C1—H1C	109.5
F3—C8—C7	113.03 (18)	H1B—C1—H1C	109.5

### Hydrogen-bond geometry (Å, °)

**Table:**

*D*—H···*A*	*D*—H	H···*A*	*D*···*A*	*D*—H···*A*
C1—H1C···F2^i^	0.96	2.52	3.292 (3)	138
C6—H6A···F3	0.93	2.35	2.696 (3)	102
N—H0A···F1^ii^	0.86	2.44	3.242 (2)	155
N—H0B···N^iii^	0.86	2.47	3.245 (3)	150

Symmetry codes: (i) *x*+1, *y*, *z*; (ii) −*x*+1/2, *y*−1/2, *z*; (iii) *x*−1/2, *y*, −*z*+1/2.
